# Primitive Neuroectodermal Tumour of Subcutaneous Tissue Presenting as a Shoulder Lump: A Case Report

**DOI:** 10.31729/jnma.6266

**Published:** 2022-03-31

**Authors:** Shankar Poudel, Upama Sangroula, Ashik Rajak

**Affiliations:** 1 Department of Radiology and Imaging, Indira Gandhi Memorial Hospital, Male, Republic of Maldives; 2 Department of Emergency Medicine, Indira Gandhi Memorial Hospital, Male, Republic of Maldives; 3 Ministry of Health, Republic of Maldives

**Keywords:** *case report*, *magnetic resonance imaging*, *neuroectodermal tumour*, *neuron-specific enolase*, *subcutaneous tissue*

## Abstract

Primitive neuroectodermal tumour is a poorly differentiated small round cell neoplasm that primarily affects children and is very rarely seen in adults. Peripheral primitive neuroectodermal tumours are rare compared to the central type and resemble soft tissue sarcoma. Primitive neuroectodermal tumours involving the subcutaneous tissue are rare and only a few cases involving the subcutaneous tissue of the anterior abdominal wall have been reported. However, no cases involving the subcutaneous tissue of the shoulder region have been reported. We report the case of a peripheral primitive neuroectodermal tumour arising from subcutaneous tissue of the right shoulder in a young adult.

## INTRODUCTION

Primitive neuroectodermal tumour is a rare family of malignancy comprising osseous Ewing sarcoma, extraosseous Ewing sarcoma, Primitive Neuroectodermal Tumour (PNET), and Askin tumour. These tumours show small round cells with varying degrees of neuroectodermal differentiation. PNET shows evidence of neuroectodermal differentiation, whereas Ewing sarcoma lacks this differentiation.^1^ The term PNET was coined by Hart and Earle in 1973, and common during the first and second decades of life.^1^ Peripheral PNET/ Askin tumours are mostly aggressive and malignant; the common site is the chest wall. They rarely involve the retroperitoneum, paraspinal region, neck, kidney, liver, cervix, lung, uterus, ovaries, penis, pancreas, urinary bladder, skin, and subcutaneous tissue.^2^

## CASE REPORT

A 30-year-old female presented to the emergency outpatient department with swelling in the right shoulder region for two months. The swelling was progressively increasing in size with no skin erythema or pus discharge. On examination, the lump was measured to be approximately 5x5 cm in size and firm in consistency. There was a full range of motion in the right shoulder joint which excluded shoulder joint involvement.

The laboratory reports were within normal limits. Ultrasound revealed a well-defined solid-cystic lesion measuring approximately 6x5 cm in size with peripheral solid components involving the subcutaneous plane of the posterior superior aspect of the right shoulder region. On the colour doppler, mild vascularity was noted in the solid component.

**Figure 1:**
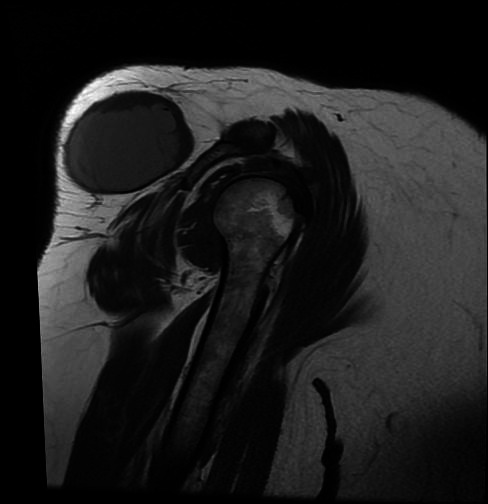
T1 weighted image sagittal section showing a well-defined solid-cystic lesion involving the subcutaneous plane of the right shoulder.

**Figure 2:**
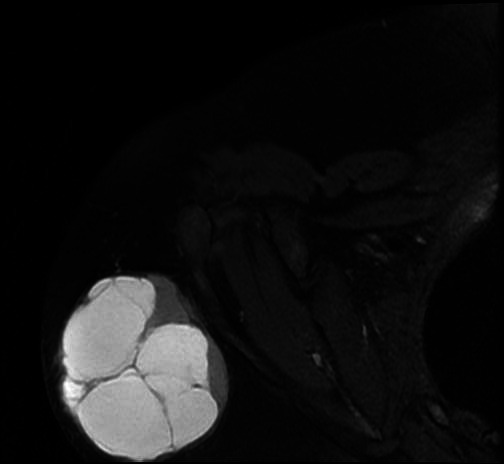
T2 fat saturation axial image showing solid-cystic lesion involving the subcutaneous plane of the posterior aspect of the right shoulder.

MRI scan showed well-defined heterogeneous predominantly cystic lesion measuring 6.9x6.8 cm involving the subcutaneous plane of the posterior superior aspect of right shoulder appearing hypointense on T1 and hyperintense on T2 with peripheral solid component appearing isointense on both T1 weighted imaging (Figure 1) and T2 FAT SAT weighted imaging (Figure 2). Multiple thin hypointense internal septations were also seen. However, a contrast study was not performed.

**Figure 3:**
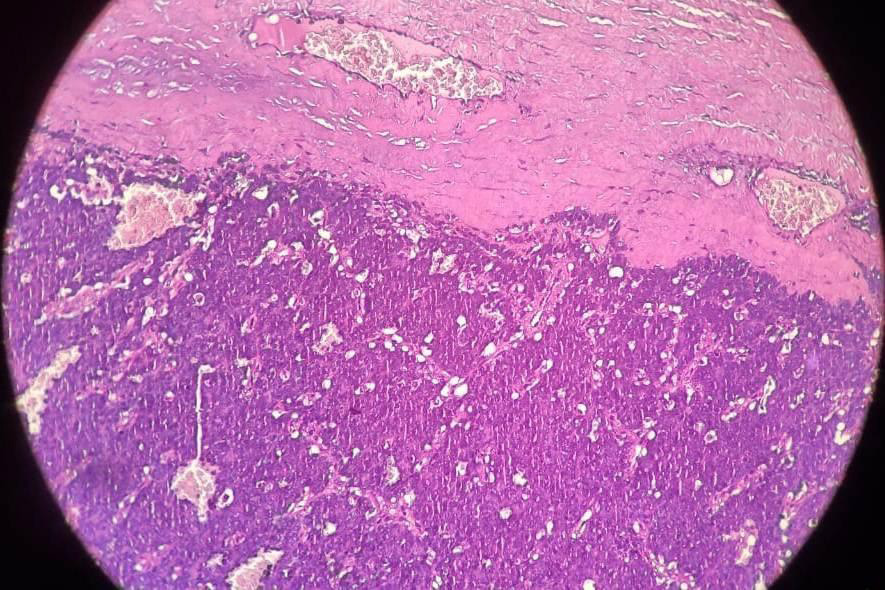
Light microscopy showing multiple small round cells with scanty cytoplasm, prominent nucleus, and capillary network.

The patient underwent excision of the cyst and it was sent for histopathological examination. Grossly, the specimen was a grey-brown multiloculated cyst measuring 6x5x4 cm. On the cut section, it showed congested solid areas with bloody fluid. The maximum cyst wall thickness was 2 mm. On microscopy, multiple sections showed lesions composed of sheets of small round cells with a prominent capillary network. These small cells had scanty cytoplasm with a round to ovoid nucleus (Figure 3).

Diagnosis of small round cell tumour was made and was sent for immunohistochemistry evaluation which showed positive Vimentin, Synaptophysin, MIC-2 (CD99), CD56, and neuron-specific enolase that suggested PNET.

## DISCUSSION

PNETs are malignant tumours of neuroectodermal origin consisting of small round cells and are classified under Ewing's sarcoma family of tumours. It is divided into central PNET involving the central nervous system and peripheral PNET. Peripheral PNETs are rarer than central PNETs and resemble soft tissue sarcoma. PNET/Ewing sarcoma family exhibit common histology phenotype, expression of MIC-2 protein (CD99), CD56, and neuron-specific enolase.^3^

Peripheral PNETs including those in the eye, maxillofacial region, peripheral limbs, gynaecological organs, intra-abdominal, skin, and subcutaneous tissue have been reported previously. However, involvement of subcutaneous tissue of the shoulder region presenting as a shoulder lump has rarely been reported.^4^ Clinically, PNET appears as a rapidly growing mass with symptoms of pain, skin erythema, and compression. It can occur at any age but children and adults are commonly affected. It can occur at any location, but the thoracic pulmonary region, extremities, head, and neck are comparatively more common. Diagnosis of PNET should be considered if tomography image shows a large, ill-defined, aggressive cystic-solid mass with persistent moderate enhancement, with or without osteolytic bone destruction.^5^

Large size tumours often show cystic changes or necrosis due to inadequate vascular supply compared to the size. MRI shows variable findings appearing iso to hypointense in T1 and iso to hyperintense in T2 weighted imaging. They show heterogeneous enhancement with areas of necrosis or cystic degeneration. Tumours usually have ill-defined borders and irregular shape.^6^ Subcutaneous PNET is frequently underdiagnosed. Correct diagnosis can be made based on histology features, demonstration of neuroendocrine granules on electron microscopy, and combination of MIC-2 ( CD99), CD56, and more than one neural marker positivity.^7^

Histologically, PNET consists of sheets of small round cells with a high nuclear to cytoplasm ratio. The cytoplasm is scanty, eosinophilic, and contains glycogen with stain PAS. Nuclei are round, with finely dispersed chromatin and a few tiny nucleoli with occasional rosette formation. The tumour often shows necrosis with viable cells on perivascular distribution. On immunohistochemistry, PNET shows a membranous expression of MIC-2 or CD99. Depending on their degree of differentiation, tumour cells express positive to neuron-specific enolase, synaptophysin, and S-100 protein. A combination of light microscopy pictures of small round cells and immunohistochemistry findings of CD99, CD56, and neuron-specific enolase help to make a diagnosis of PNET.^8^

There are many tumours in the small round cell family showing positive CD99 and PNET as one of the members of the small round cell tumour family should be considered a differential diagnosis in case of any soft tissue tumour. Furthermore, cells must demonstrate the panel of immunohistochemistry to reach a definite diagnosis.
